# Fluorescent Protein-Based Indicators for Functional Super-Resolution Imaging of Biomolecular Activities in Living Cells

**DOI:** 10.3390/ijms20225784

**Published:** 2019-11-17

**Authors:** Kai Lu, Cong Quang Vu, Tomoki Matsuda, Takeharu Nagai

**Affiliations:** 1 The Institute of Scientific and Industrial Research, Osaka University, Ibaraki 567-0047, Japan; ka1@sanken.osaka-u.ac.jp (K.L.);; 2 Graduate School of Frontier Biosciences, Osaka University, Suita, Osaka 565-0871, Japan

**Keywords:** fluorescent protein, GFP, photochemistry, phototransformation, genetically encoded indicator, BiFC, PAINT, FRET, super-resolution microscopy, nanoscopy

## Abstract

Super-resolution light microscopy (SRM) offers a unique opportunity for diffraction-unlimited imaging of biomolecular activities in living cells. To realize such potential, genetically encoded indicators were developed recently from fluorescent proteins (FPs) that exhibit phototransformation behaviors including photoactivation, photoconversion, and photoswitching, etc. Super-resolution observations of biomolecule interactions and biochemical activities have been demonstrated by exploiting the principles of bimolecular fluorescence complementation (BiFC), points accumulation for imaging nanoscale topography (PAINT), and fluorescence fluctuation increase by contact (FLINC), etc. To improve functional nanoscopy with the technology of genetically encoded indicators, it is essential to fully decipher the photo-induced chemistry of FPs and opt for innovative indicator designs that utilize not only fluorescence intensity but also multi-parametric readouts such as phototransformation kinetics. In parallel, technical improvements to both the microscopy optics and image analysis pipeline are promising avenues to increase the sensitivity and versatility of functional SRM.

## 1. Introduction

The advent of fluorescent proteins (FPs) is a unique case of biotechnology development and a manifestation of “seeing is believing.” The jellyfish *Aequorea Victoria* green fluorescence protein (GFP) was first discovered many decades ago, and its gene was cloned in the 1990s. The evolutionary significance and biological function of this peculiar protein were all but a mystery at the time (it was later found that GFP can be a light-induced electron donor, etc.) [1]. Nevertheless, the fluorescence of GFP proved to be highly utilitarian for in situ labeling of cells and proteins [2,3]. Thus the hunt for new FP templates in light-emitting organism began, and mutations that improve fluorescence properties were actively sought after via molecular evolution in laboratories. Today, the FP family has expanded to cover the light spectrum from ultraviolet (UV) to near infrared, bearing fruits of bright and environmentally stable FPs suitable for various bioimaging applications [4]. Although competing technologies of fluorescent labeling kept emerging, the popularity of FPs has never diminished. Compared to chemical dyes and quantum dots, FPs can be genetically encoded, i.e., expressed within target cells as transgenes. The versatility brought forward by genetic encoding is not to be understated. First and foremost, intracellular proteins can be easily visualized in living cells through FP fusion. Beyond that, FP expression can be toggled with inducible promoters, e.g., via the tetracycline (Tet)-controlled expression system. Endogenous proteins can be labelled with genome editing tools such as zinc finger nucleases, transcription activator-like effector nuclease (TALEN) and clustered regularly interspaced short palindromic repeats (CRISPR). Gene activation or silencing can be monitored with tissue-specific or pathologically regulated promoters, etc. Although some fluorescent dyes can be ligated to genetically encoded peptides (e.g., Halo-, SNAP- and CLIP-tags), caveats such as nonspecific binding, cytotoxicity, and poor ligand permeability pose challenges for live-cell imaging applications [5].

Apart from using FPs as light-emitting labels, it is possible to engineer genetically encoded indicators to report biomolecular activities with fluorescence changes. In 1997, Miyawaki et al. developed a chimeric protein that fluoresces in response to intracellular calcium ions [6]. To enable calcium imaging, a Ca^2+^-binding calmodulin-M13 hybrid domain is sandwiched strategically between two GFP-derived FPs that form a Förster resonance energy transfer (FRET) pair. Ca^2+^ binding switches calmodulin-M13 from an extended dumb-bell-like form to a compact globular form. This conformational change pulls donor and accepter closer together and elevates the FRET. The advent of this genetically encoded Ca^2+^ indicator opened the new avenue of visualizing bimolecular activities in living cells. Since then, over 700 genetically encoded indicators have been developed for detecting protein behaviors and various biochemical activities, with many potent designs being brought to the table over the years. This review does not offer itself as a shortcut to the enormous goal of learning all these indicators. Neither does it aims for something as ambitious as excellence in the rational engineering of FPs and indicators. For that audience, comprehensive reviews on genetically encoded indicators exist elsewhere [7,8]. Instead, we aim for the eminently possible goal of understanding in a much more definite niche: namely the interface between FP-based indicators and super-resolution light microscopy (SRM).

Genetically encoded indicator technology is arguably an indispensable part of microscopy, inasmuch as biomolecular activities must be revealed in the context of cellular architecture to stay relevant. Most off-the-shelf indicators are designed with conventional fluorescence microscopy in mind and are targeted for ensemble imaging. In consequence, spatial resolution of the optical systems is limited by the physical law of light diffraction and thus caps around half of the fluorescent emission wavelength [9]. Accumulating evidence has made it increasingly clear that intracellular signaling is often segregated into discrete nanoscopic domains. Sensing biological events on such a minute scale demands improved spatial resolution of microscopes. To this end, functional SRM began to receive attentions recently [10]. Unlike canonical SRM, which aims to reveal ultrafine structural details, functional SRM attempts to extract information regarding intracellular environments and physiology from the multi-parametric super-resolved fluorescence signals. However, early implementations of functional SRM were mostly restricted to biophysical investigations of polarity [11,12,13] and hydrophobicity [14], etc., rather than focusing on the biology of living cells. The imaging experiments also rely heavily on fluorescent dyes [12,13,14], thus lack the many advantages of genetically encoded systems. This review serves as a status report that is dedicated to the newly formed alliance between FP-based indicators and SRM, toward revealing not only nano-architecture but also nano-sequestered biomolecular activities in living cells. In particular, we focus on imaging techniques that exploit phototransformation behaviors of FPs, including photoconversion, photoactivation, and photoswitching [4]. We will lay down the immediate challenges of developing indicators for diffraction-unlimited microscopy, provide examples of monitoring biomolecule interactions and biochemical activities in nanoscopic domains, and discuss how phototransformation has been exploited in unprecedented manners for bioimaging innovations transcending spatial resolution improvement. Potential target audiences are adventurous FP engineers and indicator developers keen to explore advanced imaging techniques, as well as microscopists who seek to expand nanoscopy beyond a structure visualization tool. A holistic understanding of indicator designs and SRM can be expected by the end of this review, with information and clues being provided for future advancements in this field.

## 2. Sense and Sensibility: Anatomy of Genetically Encoded Indicators

In this review, genetically encoded indicators are defined as FP-based chimeric proteins exhibiting fluorescence sensitized to physiological or biomolecular cues. A typical indicator consists of two core elements: a sensing domain and a fluorescent reporter. The sensing domain is usually derived from endogenous proteins or peptides that are inherently sensitive towards the biological activity of interest. In the case of calcium indicators, calmodulin from the calcium signal transduction pathway is often used for recognizing calcium ions through its EF hand motifs [6,15,16]. The Ca^2+^-associated holo-calmodulin undergoes a conformational change and relays the information to the fluorescent reporter. Thus, calcium concentration is converted to fluorescence signals that enable microscopic observation. To sensitize the fluorescent reporter moiety for activity-dependent response and maximize dynamic range of the indicator, FPs are often subjected to further configuration such as circular permutation (cp) [16,17], reconstitution of split fragments [18], and FRET pairing [6,19]. Notably, chromophores of many FPs are sensitive to factors like pH, temperature and ionic strength [20]. While such environmental sensitivity is normally deleterious for ubiquitous fluorescence imaging, it nevertheless becomes an exploitable feature for sensing physiological conditions inside the cell. In such applications, FPs are not only the passive reporter but also double in the active role of sensing domain. One example is the development of a pH-sensitive FP, pHVenus, by introducing a H148G mutation to the yellow FP Venus [21] and therefore intentionally unshielded the chromophore by creating a solvent channel in the cavity for higher p*K*_a_, i.e., higher pH sensitivity [22]. For general fluorescence labeling, high pH sensitivity of FPs is undesirable and avoided. However, pHVenus found its niche and has been successfully used as an indicator for intracellular pH when expressed in living cells [22]. Indicators like pHVenus highlight the importance of tuning fluorescence properties for specific applications in a context-dependent manner, instead of judging the merit of FPs with a universal standard.

Besides the sheer efforts from protein engineers to continuously update the indicators, convergent evolution with microscopy is essential for expanding the possibility of this FP-based technology. Historically, epifluorescence and confocal microscopies have become the primary tools for general bioimaging purposes. However, spatial resolution of conventional microscopy is bound by Abbe’s diffraction limit [9] which has since been surpassed by SRM. Regional biomolecular activities in cells tend to be both minuscule and transitory. Unsurprisingly, some fleeting phenomena are not detected by ensemble imaging. SRM provides an opportunity to lift this barrier and catch singular events which could potentially impact the overall cell function.

The most direct approach for nanoscopic observation of biomolecule activities is perhaps to reach out for the huge back catalogue of indicators. Among SRM techniques, stimulated emission depletion (STED) and structured-illumination microscopy (SIM) are, in principle, directly backward compatible with preexisting indicators made of constitutive FPs. STED excites a diffraction-limited spot but uses a second laser to deplete emission within an Airy disk, therefore producing a refined subdiffraction illumination area [23]. The final STED image is obtained by scanning a sample with the dual lasers (as opposed to common point scanning seen in confocal microscopy) that effectively narrows point spread function (PSF) of the fluorophores. Intensity information from emission, i.e., the indicator’s quantitative readout, is well preserved in STED; as a result, migration from diffraction-limited microscopy to STED is straightforward in theory for imaging conventional indicators. On the down side, STED depletion beam deploys some of the highest laser power among all SRM techniques, raising concerns over phototoxicity to biological samples and unrecoverable photodestruction of FPs (phototoxicity and FP photodestruction are often loosely quoted under the collective term of photodamage). Despite that, time-lapse STED imaging in living cells has been demonstrated on several occasions [24,25]. For functional SRM, a H_2_O_2_ indicator based on cpYFP, named HyPer2, has been successfully observed with STED [26]. Although systematic studies of photodamage remain criminally uncommon, smart light-dose engineering could potentially be a game changer for live-cell STED, e.g., fast laser scanning and reduced dwell time allow living samples to recover viability during dark breaks of acquisition [27]. In addition, red-shifted fluorophores are attractive options for STED because of drastically reduced phototoxicity by up to several hundred folds over FPs excited with UV or blue light [28]. These FPs are usually derived from phytochromes or cyanobacteriochromes. They bear novel external bilin chromophores, which is a departure from the β-barrel scaffold of GFP. Recently, effective brightness and monomeric property of near-infrared biliproteins have been greatly improved to accommodate bioimaging applications in mammalian cells [29,30,31]. For SRM, a photostable biliprotein with far-red emission, named SNIFP, has been successfully applied to the imaging of cytoskeleton and nucleus pores in living cells (notably, low quantum yield of SNIFP is likely offset by the combination of strong laser excitation in STED and sensitive detector) [25]. Compared to STED, SIM requires less aggressive illumination scheme, and is arguably more amiable towards living samples [32]. However, standard SIM is ultimately still bound by the physics law of diffraction, so the maximum resolution improvement is only two-fold over the diffraction limit. In additional, obtaining the final SIM image requires mathematical reconstruction from raw data, a complication compared to the direct output of STED.

## 3. A Tale of Two Cities: Phototransformable Indicators for Super-Resolution Fluorescence Microscopy

Except for STED and SIM, phototransformation of fluorophores are exploited in many other SRM methods, such as photo-activated localization microscopy (PALM) [33,34], super-resolution optical fluctuation imaging (SOFI) [34,35], super-resolution radial fluctuations (SRRF) [36], Bayesian analysis of the blinking and bleaching (3B) [37], entropy-based super-resolution imaging (ESI) [38], spatial covariance reconstructive (SCORE) [39], and multiple signal classification algorithm (MUSICAL) [40]. To ease non-microscopists out of this perplexing jungle of acronyms, these techniques universally separate emitters by photo-inducing fluorophores to stochastically traverse two or more distinctive states, then approximate emitter positions through mathematical calculation, and reconstruct the super-resolved image. Notably, these techniques have different upper limits for the density of on-state fluorophores. For PALM, sparsely photoactivated fluorophores must be separated by a distance greater than the diffraction limit, thus requires strict single-molecule blinking; though some multi-emitter fitting algorithms now allow certain degree of fluorophore overlapping in single-molecule localization microscopy (SMLM), e.g., the DAOSTORM algorithm [41]. Compared to SMLM, other nanoscopies have relaxed requirement of emitter density and output super-revolved images from fluorophore fluctuation or flickering data, e.g., SOFI, SRRF and 3B. An excellent introduction of SRM can be found in a timely review by Schermelleh et al. [42], thus out of the scope of this paper. Although photochromism may also be induced in certain constitutive FPs in the presence of chemical cofactors, e.g., incubating mCherry with thiol or β-mercaptoethanol leads to reversible fluorescence off or red-to-blue emission [43,44], the most non-invasive and convenient approach to introduce blinking in living cells is still by using phototransformable FPs [45,46,47]. Light-induced transformation phenomena that are commonly exploited in SRM include photoactivation [48,49], photoconversion [50,51], and reversible photoswitching [52,53,54]. Although current understanding of FP photochemistry is far from complete, it is most likely that for the GFP family, *cis*-*trans* isomerization of the chromophore coupled to protonation state changes, plays a key role in the process [55].

The majority of preexisting indicators rely on fluorescence intensity to readout the biomolecular activity of interest. However, in super-resolution imaging experiments, accurate measurement of intensity is often obscured. First, conventional microscopy takes ensemble fluorescence, which is the combined signals from many emitters and of high signal-to-noise ratio (S/N), whereas single-molecule imaging ought to work with much smaller photon budget and lower S/N, making intensity measurement especially sensitive to unevenness in background. To improve this situation, confined illumination with total internal reflection fluorescence (TIRF), highly inclined and laminated optical sheet (HILO) or light sheet are now commonplace. Red-shifted illumination also helps to reduce autofluorescence. More often than not, these tricks remain insufficient for accurately knowing the intensity of individual emitters in biological samples. Moving forward, elaborated methods for the computation and subtraction of local background on single-molecule level, e.g., SMALL-LABS, may help remove systematic bias in gauging fluorescence intensity on nanoscale [56]. Second, as a pitfall of post-acquisition image reconstruction, intensity of computational SRM often deviates from the typical linear response, e.g., non-linear brightness is introduced by high-order cumulant statistics in SOFI. While such sacrifice is necessary for breaking the diffraction limit, it is expected to squish the dynamic range of indicators and undermine data interpretation. Improved imaging reconstruction algorithms have emerged to relieve this issue, e.g., balanced SOFI combines information from cumulants of several different orders to linearize the fluorescence response [57], but wide adoption of such techniques in functional SRM remains uncommon, partly because of the technical complexity.

It is evident that developing indicators for SRM requires appreciation of the principles of diffraction-unlimited imaging, thus cannot be brutally forced with prior routines. While intensity-based indicators are pending a breakthrough, super-resolution biosensing has otherwise been successfully demonstrated by circumventing this limitation with nifty designs (summarized in Table 1).

### 3.1. Diffraction-Unlimited Observation of Protein–Protein interactions by Bimolecular Fluorescence Complementation (BiFC) of Split Phototransformable Fluorescent Proteins

**Figure 1:**
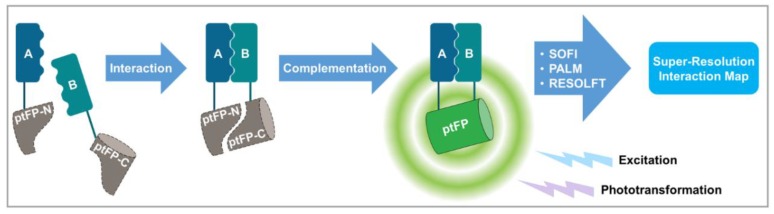
Principle of super-resolution imaging of protein-protein interactions with BiFC of phototransformable fluorescent proteins (ptFPs). Protein A is fused to an N-terminal fragment of the ptFP (ptFP-N); and protein B is fused to the complementary C-terminal fragment (ptFP-C). When protein A and B interacts, ptFP-N and ptFP-C reconstitute. Following chromophore maturation, fluorescently labeled interaction loci can be excited and phototransformed for super-resolution detection with SOFI, PALM, or RESOLFT. The choice of SRM technique depends on photoactivation, photoconversion, or photoswitching property of FP used in each study.

To achieve diffraction-unlimited imaging without radical overhaul of available indicator designs, bimolecular fluorescence complementation (BiFC) has quickly become a popular strategy. These indicators are typical for detecting protein-protein interactions (PPIs), but their functionality also extends to the imaging of biochemical activities. In BiFC system, a FP is split into two parts, namely an N-terminus fragment and its complimentary C-terminus counterpart. Neither fragments emit light independently. To develop indicators, interacting proteins of interest are fused to each FP fragments separately. When PPI occurs, the two fragments converge and undergo molecular complementation. After chromophore maturation, the reconstituted FP fluoresces and facilitates the visualization of interactions (Figure 1). Compared to indicators that rely on analogue intensity, BiFC indicators have binary readout—the two states, namely fluorescent or non-fluorescent, correspond to the presence or absence of PPI respectively.

To enable super-resolution imaging of BiFC, fluorescent reporter moiety of the indicators is switched from a constitutive FP to a phototransformable one (Figure 1). From the view point of protein engineering, the main challenge is to experimentally identify split site for each phototransformable FP with hints from the protein structure. Importantly, FPs must regain both fluorescence and phototransformability after bimolecular complementation. To date, BiFC split sites have been reported for several phototransformable FPs including cpDMVF (a circular-permutated mutant of the reversibly photoswitchable FP Dronpa) [58], PAmCherry1 [59], mEos3.2 [60], PA-GFP [61], mIrisFP [62], and rsEGFP2 [63]. While only one split site was identified in most cases, two split sites at a.a. 150 and a.a. 165 were reported for mIrisFP, which enables the detection of three proteins forming a complex (Three-Fragment Fluorescence Complementation, i.e., TFFC, is an extension of the BiFC technology) [62]. For proof of concept, mIrisFP-based TFFC system was applied to the heterotrimer of G_s_ protein which contains α_s_, β_1_, and γ_2_ subunits. In combination with PALM microscopy, signal-dependent dissociation of α_s_ subunit from the β_1_ and γ_2_ heterodimer was revealed [62].

Despite the hassle of finding functional split sites for phototransformable FPs, the downstream super-resolution observation of PPIs is relatively straightforward. Sample preparation is identical to conventional BiFC, and standard SRM methods are adopted without major modification to the imaging protocols. The technique branches into three categories based on the choice of SRM module: refSOFI (i.e., BiFC-SOFI) [58], BiFC-PALM [59,60,61,62], and BiFC-RESOLFT [63]. The main difference among these implementations is the particular combination of achievable spatial and temporal resolutions. For instance, localization precision of BiFC-PALM is as high as tens of nanometers, but acquisition of each PALM image can take up to minutes. refSOFI is faster because larger proportion of fluorophores stay in fluorescence-on state and smaller number of raw frames are required for reconstructing each SOFI image, but spatial resolution is around 100 nm and shies away from SMLM. Reported BiFC-based phototransformable indicators and their uses are summarized in Table 1. Nevertheless, BiFC-based indicators come with a few limitations. First, kinetics of these indicators is slow. There is a lag between interaction occurrence and the onset of detectable fluorescence, with chromophore maturation being the rate-limiting step. Depending on maturation speed of each FP, the delay may range from several minutes to hours. Second, BiFC is mostly an irreversible process. After BiFC occurs, the associated status cannot be reverted. Although mostly a nuisance for observing dynamic dissociation and reassociation, the irreversibility does bring an unexpected benefit for SRM practice. Because the complemented status of indicator molecule is locked-in, transient PPIs that would otherwise escape real-time detection will leave behind a stable fluorescent signature. This make post-hoc detection possible and the use of SRM techniques of slow temporal resolution a practical option. Notably, as an alternative to the BiFC of β-barrel type FPs, splitFAST, a reversible split fluorescent reporter was developed recently based a phytochrome FP variant that binds a fluorogen [64]. The splitFAST system consists of two split fragments of a 14 kDa photoactive yellow protein (PYP) mutant derived from *Halorhodospira halophila*, which recruit hydroxybenzylidene rhodanine (HBR) analogs as external chromophores after protein complementation. The FP-fluorogen complex is reversible and therefore overcomes one limitation of traditional BiFC for monitoring dynamic interactions.

### 3.2. Diffraction-Unlimited Observation of Biomolecule Interactions by Points Accumulation for Imaging Nanoscale Topography (PAINT) with Photoconvertible Fluorescent Proteins

**Figure 2:**
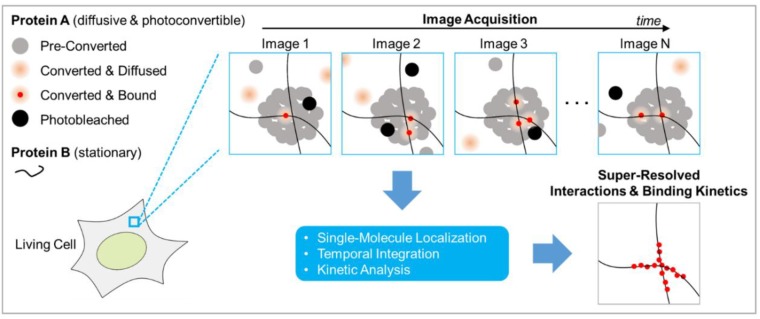
Principle of super-resolution imaging of biomolecule interactions with PAINT-PALM. For illustration purpose, the interactions between a diffusive protein A and a stationary protein B are shown here. The concept is readily generalizable to the imaging of protein-DNA or protein-RNA interactions in the nucleus, by replacing protein B with DNA or RNA in the figure. Here, the diffusive protein A is fused to a photoconvertible fluorescent protein (FP) and expressed in living cells. During experiment, the chimeric protein A (ligand) is stochastically photoconverted to fluoresces. On camera, the images of unbound protein A molecules appear motion-blurred because of diffusion. The images of protein A molecules interacting with protein B appear as sharp spots with well-defined Gaussian PSF, after transient immobilization. The interactions are sampled over time by acquiring an image sequence (image 1, 2, 3, …, *N*). Super-resolved interaction map is generated by single-molecule localization and temporal integration. The temporal image sequence may also be subjected to additional analysis such as tcPALM to extract information on binding kinetics.

The migration of BiFC-based PPI imaging from diffraction-limited to SRM feels more like an upgrade than a completely new invention. By breaking away from the standard indicator design philosophy and tapping into the fundamentals of single-molecule imaging, points accumulation for imaging nanoscale topography (PAINT) has been repurposed for imaging biomolecule interactions at super resolution (Table 1). To distinguish itself from PALM, PAINT relies on binding of fluorescent ligands to stationary targets, rather than stochastic photoactivation of permanently bound fluorophores to generate single-molecule blinking. In its original form, PAINT is performed with Nile Red dye which is transiently immobilized when colliding with the sample surface [65]. Later, specificity of PAINT in fixed cells was boosted by using complementary DNA strands to induce transient bindings (DNA-PAINT) [66]. For functional SRM of PPIs, the concept of PAINT has been generalized to the genetically encoded system of FPs. To illustrate the idea behind PAINT-based PPI detection, imagine a case of investigating the interaction between a diffusive protein A and stationary protein B (Figure 2). Here, protein A is fused to an FP, and protein B serves as the bait. In the absence of interaction, protein A molecule diffuses freely, and its image is motion-blurred and dim. Upon interacting, protein A docks to protein B, and image of the transiently immobilized protein A now appears as a bright and sharp dot. With appropriate camera exposure, only the subpopulation of protein A molecules that interact with protein B assume a well-defined Gaussian PSF. Therefore, only interacting molecules are localizable and contribute to the reconstructed super-resolution image (Figure 2).

For example, plasma membrane binding of cytosolic proteins that are involved in the epidermal growth factor (EGF) signaling pathway was investigated with this approach. A photoconvertible FP mEOS3.2 was fused to Grb2, c-Raf, or PLCγ1, respectively, and used as PAINT probes for membrane anchorage [67]. By combining PAINT with the green-to-red photoconversion of mEOS3.2 (PAINT-PALM), single-molecule imaging unraveled a membrane heterogeneity during spatiotemporal regulation of EGF signaling. Beyond PPI detection, this method was also applied to protein–DNA interaction and protein–RNA interaction in the nucleus. With a photoconvertible FP mEOS3.1, DNA binding of a replication licensing factor, Mcm4, and a processivity factor for DNA polymerase δ, PCNA, were imaged at various stages of the cell cycle in living fission yeast [68]. For protein–DNA interaction in living mouse embryonic stem cells, the transcriptional factor Sox2 was fluorescently labeled with TMR dye via HaloTag. And association kinetics of Sox2 with chromatin was interrogated. Although in this study, TMR dye was the fluorescent ligand of HaloTag and the system was only partially genetically encoded [69]. For protein-RNA interaction, RNA polymerase II (Pol II) was labeled with Dendra2, an FP that converts from green to red emission upon UV illumination. Following PAINT, temporal clustering of Pol II in living mouse embryonic fibroblasts was quantified by time-correlated PALM (tcPALM). tcPALM is novel method for analyzing binding kinetics at super-resolution, and it was used here to estimate relative local concentrations of interacting protein from changes in the frequency of single-molecule detections [70,71].

Compared to the irreversibility of BiFC, diffusive PAINT probes are constantly replenished from the surrounding environment. This means that transient interactions can be sampled repeatedly, despite the fact that probes are irreversibly photoconverted or photobleached. Again, compared to BiFC, which must wait for chromophore maturation, kinetics of PAINT imaging is fast. The real-time detection takes only tens to hundreds of milliseconds, which allow highly transient interaction to be monitored. Notably, phototransformation is not strictly required for PAINT-based functional SRM, providing interactions are already rare by nature and appear sparsely in snapshots (fewer than one fluorescent molecule per diffraction-limited volume). In practice, association affinity is high for many biomolecule interactions under physiological condition, which leads to densely populated fluorescence signals and low S/N. The introduction of photoconversion to PAINT, i.e., PAINT-PALM, helps break this concentration barrier by suppressing the density of on-state fluorophore and therefore allows for burst of interactions with high affinities to be super-resolved [72]. Further down the road, it might be possible to incorporate the concept of single-molecule photoactivation FRET (sm-PAFRET) to improve the range of detectable concentration with PAINT-based super-resolution interaction imaging [73].

Before moving on, a note should be taken for estimating spatial resolution in functional SRM. For single-molecule imaging such as PAINT-PALM and BiFC-PALM, localization precision is often reported (Table 1). Localization precision, i.e., optical resolution of the imaging system, only reflects how accurate the instrument can determine the position of single molecules. For conventional SRM that aims at resolving ultrafine structural features of, e.g., filaments and focal adhesions, localization precision alone can be insufficient and misleading for describing the revolving power [74,75]. Instead, structural resolution should also be measured by full width at half maximum (FWHM) or Fourier ring correlation (FRC) [76], etc. For functional SRM of PPIs, however, the precise locations of interactions and binding kinetics are arguably more informative than pure structural information. Thus the choice of reporting localization precision as the performance indicator of spatial resolution is somewhat justified in these cases of functional SRM.

### 3.3. Diffraction-Unlimited Observation of Biomolecular Activities Based on Fluorescence Fluctuation Increase by Contact (FLINC)

While biomolecule interactions including PPIs, protein-DNA interactions, and protein-RNA interactions can be super-resolved with BiFC-type or PAINT-type indicators, diffraction-unlimited observation of biochemical activities has been elusive until very recently. As an unconventional phototransformation phenomenon, fluorescence fluctuation increase by contact (FLINC) broke this silence by using fluorophore blinking behavior as a quantitative readout [77]. FLINC is the unexpected discovery of fluorescence fluctuations of TagRFP-T in physical approximation to Dronpa, a reversely photoswitchable FP. TagRFP-T is generally considered a constitutive FP lacking obvious photoactivation, photoswitching, or photoconversion behavior. However, TagRFP-T displays elevated degree of fluctuations as its distance to Dronpa shortens. Because the extent of fluctuations can be quantified at subpixel level on SOFI image with high-order cumulant statistics, indicators that convert biochemical activities to distance change between TagRFP-T and Dronpa were developed. To this end, a FLINC-AKAR1 indicator for protein kinase A (PKA) activity was made first for proof of concept. PKA dynamics was monitored with nanometer precision on intensity-normalized SOFI images, i.e., activity maps. Apart from detecting biochemical activities, FLINC was also generalized for the detection of PPI (Table 1). By fusing TagRFP-T and Dronpa to each interacting component, respectively, an intermolecular FLINC system was developed for rapamycin-inducible dimerization between the FK506-binding protein (FKBP) and FKBP-rapamycin binding domain (FRB). Following a similar strategy, a bimolecular version of FLINC-AKAR1 was also developed by splitting the indicator into two components—FHA1-Dronpa and PKA-substrate-TagRFP-T—for detecting the weak interaction between FHA1 and phosphosubstrate.

**Figure 3:**
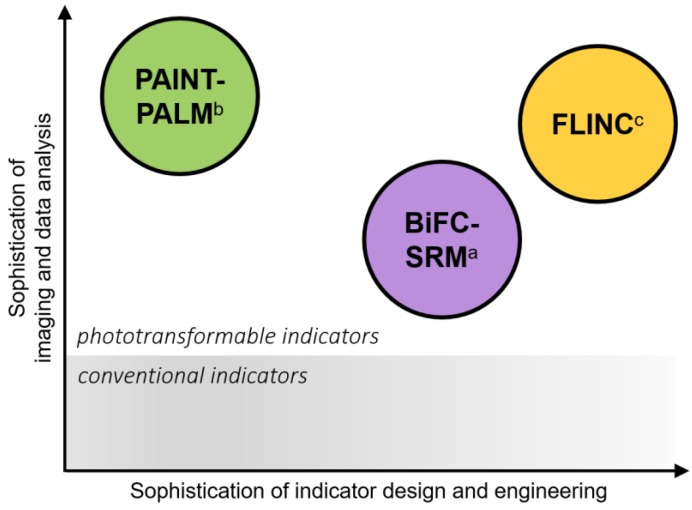
Accessibility of genetically encoded indicators derived from phototransformable fluorescent proteins for functional super-resolution imaging. a: BiFC-based indicators for SRM (BiFC-SRM) borrow the design basics from conventional BiFC indicators. The main challenges for engineering these indicators are two-folds: The first is to identify split site(s) that permits reconstitution of fluorescence and phototransformation; The second is fast chromophore maturation after complementation. Methods for SRM are straightforwardly adopted from SOFI, PALM, or RESOLFT with little modification. b: PAINT-PALM indicators are simply engineered by fusing a phototransformable FP to the diffusive protein of interest. For SRM of biomolecule interactions, both camera exposure and single-molecule localization algorithm must be fine-tuned. The goal is to filter out unbound and motion-blurred emitters (fail if exposure is too short and inclusion criteria set too low); in the meantime, bound emitters must assume well-defined Gaussian PSFs to be successfully localized (fail if exposure is too long and inclusion criteria set too high). c: Design of FLINC indicator is generalizable to the detection of both biochemical activities and PPI. Main limitation is the incomplete mechanistic understanding of FLINC phenomenon, which limits the discovery of new FLINC protein pairs. To generate super-revolved map of biomolecular activities, cumulant values must be properly normalized to eliminate bias from uneven distribution of indicators across the cell.

It is noteworthy that FLINC is independent of either Dronpa emission or photoswitching, as demonstrated by TagRFP-T fluctuations inflicted by a non-fluorescent Dronpa mutant [77]. In that sense, the mechanism of FLINC is unique and probably akin to Dronpa-assisted disturbance to the local chromophore environment of TagRFP-T. Before a complete mechanistic understanding is drawn, it is challenging to rationally design new FLINC pairs other than the TagRFP-T-Dronpa duo. Regardless, it will be exciting to see other indicators that exploit unexpected photophysics of FPs in the future. As a note of summary, the barriers of entry or accessibility of BiFC, PAINT and FLINC indicators for functional SRM are compared in Figure 3.

## 4. Through the Looking Glass: Exploiting Phototransformation of Fluorescent Proteins for Improved Bioimaging and Biomolecular Sensing

It is perhaps apparent by now that FP phototransformation can turn out to be a valuable trait for bioimaging [55]. In fact, weak on and off blinking of GFP was documented at least two decades ago [78]. For a long time, blinking was treated as a nuisance, for it leads to unstable signals in ensemble imaging. That is until SRM finally leveraged upon it and revolutionized fluorescence imaging. Since then, FPs have been actively engineered to introduce favorable phototransformation properties such as faster photoswitching speed and high on/off contrast.

Apart from being the cornerstone of many SRM techniques, phototransformation also brings extra benefits to fluorescence imaging and opens up new possibilities for indicator engineering. To reduce photodamage in STED microscopy, RESOLFT swaps constitutive FPs for reversely photoswitchable ones and achieves sizable reduction in depletion laser power [79]. The end result is higher biocompatibility for cell and in vivo imaging. In the same fashion, phototoxicity may also be suppressed by using photoswitching FPs in other SRM techniques that involves STED beam, e.g., the use of a reversely photoswitchable FP, Kohinoor, in SPoD-ExPAN microscopy dramatically reduced the illumination laser power from 1.7 MW/cm^2^ to ~1 W/cm^2^ [80,81].

Another example of photodamage reduction is found in photoswitching FRET (psFRET) [82]. Compared to the traditional photobleaching FRET that measures donor photobleaching kinetics (not to be confused with acceptor photobleaching), psFRET uses a reversely photoswitchable FP as donor and infers FRET efficiency from photoswitching speed or fluorescence lifetime. Because acceptor absorption introduces an alternative energy transfer pathway that competes with photoswitching, slower switching off kinetics of the donor and longer lifetime are expected when FRET occurs. Here, photochromism not only leads to less aggressive illumination, but also make FRET measurement reversible since donor is temporally turn off, rather than permanently bleached out. For proof of concept, a FRET pair of Dronpa and mCherry was established. Widefield psFRET imaging of PPI and biochemical activity were demonstrated respectively with the observation of histone 2B compaction and a caspase indicator [82].

For developing single-FP indicators, an ambitious concept is to directly encode information of biomolecular activities within phototransformation. Remarkably, a unique single-FP Ca^2+^ indicator named CaMPARI was reported recently, in which photoconversion of mEOS2 is driven by the binding of calcium ions to calmodulin [83]. mEOS2 is a photoconvertible FP that switches emission from green to red under UV illumination [84]. From the template of Ca^2+^ indicator GCaMP, the fluorescent reporter cpEGFP is replaced by circularly permutated mEOS2, followed by mutagenesis-based molecular evolution. The result is a molecular switch that couples photoconversion to Ca^2+^ concentration. During calcium imaging, CaMPARI is first potentiated by UV light. The indicator changes emission color from green to red in proportion to Ca^2+^ concentration. Because photoconversion of mEOS2 is irreversible, the red/green emission ratio keeps a permanent record of total calcium flux during UV illumination. Meanwhile, S/N is also boosted by the integration of fluorescence signals over time. Therefore, CaMPARI is advantageous for recording weak and transient Ca^2+^ flux and has been applied to calcium imaging in neuronal system [83,85].

Finally, performance of phototransformable FPs can be improved without introducing extra mutations to their protein sequences. This is usually achieved through a fusion partner that modulates fluorescence properties of the FPs. For example, photostability of a photoconvertible FP, mEOS3.2, was increased two-fold, after the ligation of Janelia Fluor 646 (JF_646_) dye via a HaloTag [86]. JF_646_ quenched the photoconverted population of mEOS3.2 by FRET and therefore competed with photobleaching pathway of the FP. As a result, the FRET-assisted mEOS3.2 stayed longer in the red emission state, thus allowed particle tracking at single-molecule level for extended periods of time [86]. In another instance, a camelid-derived single-domain antibody, i.e., nanobody, was fused to a reversely photoswitchable FP, rsGreens [87], to enhance its molecular brightness, pH stability, and switching property [88]. Nanobody fusion has been previously documented to modulate the chromophore environment of GFP family FPs [89]. Dimerization, i.e., the intermolecular binding between GFP and nanobody, was implied as a mechanism for the enhanced FP properties [90].

## 5. Conclusions and Perspectives

We are witnessing a coming of age of the discipline of genetically encoded indicators, as designs of indicators begin to consolidate for applications that evoke ensemble imaging. Historically, it was the invention of FP toolbox that propelled the field to its current flourishing state. It is encouraging to anticipate that phototransformable FPs could lay the foundation for a renaissance with functional SRM. Indeed, the young field has recently begun to gain its momentum, and many inspirations can be drawn from the success of super-resolution BiFC, PAINT, and FLINC indicators. On the flip side, there are also lessons to be learnt. While conventional microscopy is ubiquitous and relatively intuitive, SRM and its image analysis pipeline remain daunting for biologists without training in optics and programming. This gap is perhaps growing wider without active dialogues and the sharing of vocabularies between microscopists and protein engineers. On the other hand, the photochemistry behind light-induced FP transformation remains to be fully understood. Without that piece of the puzzle, the rational design of new indicators is largely stalled, as exemplified by the rarity of indicators like FLINC. In summary, the functional SRM of biomolecular activities is rapidly becoming a highly interdisciplinary topic. A joint force of FP engineers, indicator developers, microscopists and programmers is expected to improve this technology for seeing the minuscule and fleeting singularities in the cellular clockwork.

## Figures and Tables

**Table 1:** Reported functional SRM studies of biomolecule interactions and biochemical activities in living cells with genetically encoded indicators.

Application	Name	Target	Class	FP	FP Config.	Split Site(s)	Microscopy	Localization Precision ^1^	FWHM ^2^	Kinetics	Reversibility	Cell Type(s)	Ref.
Rapamycin-induced FKBP and FRB interaction	refSOFI	Protein-protein interaction	BiFC	DMVF	cp; split	a.a. 181	SOFI	N/A	~100 nm	Slow ^3^	No	HeLa	[58]
Interaction of receptor tyrosine kinases HER2 & 3	refSOFI	Protein-protein interaction	BiFC	DMVF	cp; split	a.a. 181	SOFI	N/A	~100 nm	Slow ^3^	No	HeLa	[58]
Interaction of ER Ca^2+^ sensor STIM1 and Ca^2+^ channel protein ORAI1	refSOFI	Protein-protein interaction	BiFC	DMVF	cp; split	a.a. 181	SOFI	N/A	~100 nm	Slow ^3^	No	HeLa	[58]
Interactions of small GTPase Ras and its effector Raf	BiFC-PALM	Protein-protein interaction	BiFC	PA-mCherry1	split	a.a. 159	PALM	18 nm	N/A	Slow ^3^	No	U2OS	[59]
Interaction of MreB and EF-Tu	BiFC-PALM	Protein-protein interaction	BiFC	mEos3.2	split	a.a. 164	PALM	12 nm	N/A	Slow ^3^	No	E. coli	[60]
Homodimerization of microtubule plus-end hub protein EB1	BiFC-PALM	Protein-protein interaction	BiFC	PA-GFP	split	not specified	PALM	23 nm	N/A	Slow ^3^	No	HeLa; MCF7	[61]
Formation of bJun/bFos complexes	BiFC-PALM	Protein-protein interaction	BiFC	mIrisFP	split	a.a. 150; a.a. 165	PALM	18 nm	N/A	Slow ^3^	No	Vero cells	[62]
Interaction among α_s_, β_1_, and γ_2_ subunits of G_s_ ternary complex	TFFC-PALM	Protein-protein interaction	TFFC ^4^	mIrisFP	split	a.a. 150 & a.a. 165	PALM	18 nm	N/A	Slow ^3^	No	Vero cells	[62]
Interaction of Bcl-xL and Bak	BiFC-RESOLFT	Protein-protein interaction	BiFC	rsEGFP2	split	a.a. 158	RESOLFT	N/A	113 nm	Slow ^3^	No	Hela	[63]
Membrane-binding of proteins in EGF signaling pathway	PAINT-PALM	Proteinprotein interaction	PAINT	mEos3.2	default	N/A	PALM	35 nm	N/A	fast	Yes ^5^	HeLa; CHO	[67]
Binding of PCNA and Mcm4 proteins to genomic DNA	PAINT-PALM	Protein-DNA interaction	PAINT	mEos3.1	default	N/A	PALM	11 nm	N/A	fast	Yes ^5^	fission yeast	[68]
Dynamics of RNA Pol II clustering at β-actin gene locus	PAINT-PALM	Protein-RNA interaction	PAINT	Dendra2	default	N/A	PALM	31 nm	N/A	fast	Yes ^5^	MEF	[71]
Protein Kinase A (PKA) activity	FLINC-AKAR1	Biochemical activity	FLINC	TagRFP-T	default	N/A	SOFI	N/A	107–179 nm	fast	Yes	HeLa; α4CHO	[77]
Extracellular signal-regulated kinase (ERK) activity	FLINC-EKAR1	Biochemical activity	FLINC	TagRFP-T	default	N/A	SOFI	N/A	160 nm	fast	Yes	HEK293	[77]
Rapamycin-induced FKBP and FRB interaction	bimolecular FLINC	Protein-protein interaction	FLINC	TagRFP-T	default	N/A	SOFI	N/A	~107–160 nm	fast	Yes	HeLa	[77]
Interaction of FHA1 and PKA phosphosubstrate	bimolecular FLINC-AKAR1	Protein–protein interaction	FLINC	TagRFP-T	default	N/A	SOFI	N/A	~107–160 nm	fast	Yes	HeLa	[77]

^1^ Localization Precision is a parameter to report optic resolution in single-molecule localization microscopy. ^2^ Full width at half maximum (FWHM) is a parameter to report structural resolution in microscopy. Separation of structural features, i.e., intensity peak-to-peak distance, is also used to report structural resolution in a few cases in Table 1. ^3^ The onset of fluorescence in BiFC and TFFC systems are rate limited by maturation speed of the chromophore. ^4^ Three-fragment fluorescence complementation (TFFC) is a variant of BiFC for detecting interactions among three protein components. ^5^ Although photoconversion of the FPs from green to red emission is irreversible, new pre-converted probes are replenished from the surrounding environment thus enable de novo binding to target molecules. a.a.: amino acid; N/A: not applicable.

## Abbreviations

a.a.	amino acid
3B	Bayesian analysis of the blinking and bleaching
BiFC	bimolecular fluorescence complementation
cp	circular permutation
CRISPR	clustered regularly interspaced short palindromic repeats
EGF	epidermal growth factor
ESI	entropy-based super-resolution imaging
FKBP	FK506-binding protein
FLINC	fluorescence fluctuation increase by contact
FP	fluorescent protein
FRB	FKBP-rapamycin binding domain
FRC	Fourier ring correlation
FRET	Förster resonance energy transfer
FWHM	full width at half maximum
GFP	*Aequorea Victoria* green fluorescence protein
HBR	hydroxybenzylidene rhodanine
HILO	highly inclined and laminated optical sheet
JF_646_	Janelia Fluor 646
MUSICAL	multiple signal classification algorithm
N/A	not applicable
PAINT	points accumulation for imaging nanoscale topography
PALM	photo-activated localization microscopy
PKA	protein kinase A
Pol II	RNA polymerase II
PPI	Protein-protein interaction
PSF	point spread function
psFRET	photoswitching FRET
ptFP	phototransformable fluorescent protein
PYP	photoactive yellow protein
RESOLFT	reversible saturable optical fluorescence transitions
S/N	signal-to-noise ratio
SCORE	spatial covariance reconstructive
SIM	structured illumination microscopy
sm-FRET	single-molecule photoactivation FRET
SMLM	single-molecule localization microscopy
SOFI	super-resolution optical fluctuation imaging
SRM	super-resolution light microscopy
SRRF	super-resolution radial fluctuations
STED	stimulated emission depletion
TALEN	transcription activator-like effector nucleases
tcPALM	time-correlated photo-activated localization microscopy
TFFC	three-fragment fluorescence complementation
TIRF	total internal reflection fluorescence
Tet	tetracycline
UV	ultraviolet
